# Hidden Hollows of the Fetal Cranium—Lacunar Skull in Isolation

**DOI:** 10.1155/crog/8926011

**Published:** 2026-09-29

**Authors:** Grace Noonan, Lucy Sheahan, Mary Sterrett

**Affiliations:** ^1^ Department of Obstetrics and Gynecology, University of California San Diego, San Diego, California, USA, ucsd.edu; ^2^ Department of Obstetrics and Gynecology, Kaiser Southern California Permanente Medical Group, San Diego, California, USA

**Keywords:** craniolacunae, lacunar skull, Lückenschädel

## Abstract

**Introduction:**

Lacunar skull, also known as Lückenschädel or craniolacunae, is a rare congenital calvarial ossification abnormality characterized by multiple lucent defects of the skull. It is most associated with neural tube defects, Chiari II malformation, craniosynostosis, and other congenital anomalies. Isolated lacunar skull, without associated structural abnormalities, has been infrequently reported, and its natural history, prognosis, and optimal clinical management are poorly defined.

**Case Presentation:**

We report the case of a female infant born at 38 weeks and 5 days′ gestation to a 37‐year‐old gravida 6 para 3033 mother following an uncomplicated pregnancy. Prenatal genetic screening and serial fetal ultrasonography demonstrated no fetal anomalies, although a bilobed heterogeneous placenta was noted. Following an uncomplicated spontaneous vaginal delivery with Apgar scores of 8 and 9, a boggy right parietal scalp abnormality was identified on routine neonatal examination. Initial skull radiographs were inconclusive. Computed tomography (CT) with 3D reconstruction demonstrated multiple asymmetric lucent calvarial defects, more pronounced within the right parietal and occipital bones, consistent with lacunar skull. Brain parenchyma, ventricles, cranial sutures, and spine were normal. Additional evaluations revealed no associated abnormalities. Diagnostic genetic testing was deferred as no syndromic features or additional congenital anomalies were identified on evaluation. Conservative management with close clinical observation was recommended. At 6 weeks of age, the infant demonstrated normal neurological examination and age‐appropriate developmental progress.

**Discussion:**

This case describes an uncommon presentation of isolated lacunar skull diagnosed postnatally in an otherwise healthy term infant with normal prenatal imaging. Unlike most reported cases, no associated central nervous system, skeletal, or syndromic abnormalities were identified following comprehensive evaluation. This report highlights the importance of distinguishing isolated lacunar skull from more common associated conditions, outlines an appropriate diagnostic evaluation, and supports conservative management with ongoing developmental surveillance in the absence of additional abnormalities.

## 1. Introduction

Lacunar skull, also referred to as Lückenschädel or craniolacunae, is a rare congenital ossification defect of the skull characterized by a thinning or fenestrated appearance primarily in the frontal, parietal, and/or occipital bones [1]. The condition presents as defects on the inner table of the cranium in the form of shallow depressions or deep cavitations that may extend to the outer surface table, varying significantly in size, shape, and number [2]. Depending on the extent of involvement, defects may be confined to the inner table of the calvarium or extend through the full thickness of the skull, resulting in palpable cranial irregularities [3].

Lacunar skull is typically identified during the neonatal period and is classically associated with neural tube defects such as spina bifida, myelomeningocele, and encephalocele, as well as with Arnold–Chiari malformation, craniosynostosis, and scoliosis [2–9]. Given these potential associations, the detection of lacunar skull warrants comprehensive diagnostic evaluation to rule out underlying or concurrent anomalies [5]. In contrast, isolated lacunar skull without accompanying congenital anomalies appears to be exceptionally uncommon, and its clinical significance remains incompletely understood.

Current data on the isolated form of lacunar skull are limited, making accurate estimations on in‐utero diagnosis, prognosis, and parental counseling difficult. In the absence of definitive evidence, clinicians face uncertainty when guiding families through decisions regarding prognosis, delivery planning if diagnosed antenatally, and neonatal outcomes. We report a case of a full‐term infant diagnosed postnatally with isolated lacunar skull via computerized tomography (CT) imaging, with no associated anatomic anomalies and normal antenatal ultrasound findings.

## 2. Patient Information

Informed consent was given by the patient for this study.

We report a case of a 1‐day‐old female neonate who was born with isolated lacunar skull. The mother was a 37‐year‐old G6P3033 female who had an uncomplicated pregnancy with normal genetic screening and unremarkable ultrasonographic findings at anatomy scan and follow up third‐trimester ultrasound. However, the placenta was noted to be bilobed with branching vessels, and the appearance of the placenta was described as “heterogeneous” during her anatomic scan (Figure 1). She also had previously undergone two prior term vaginal deliveries with healthy neonates.

**Figure 1:**
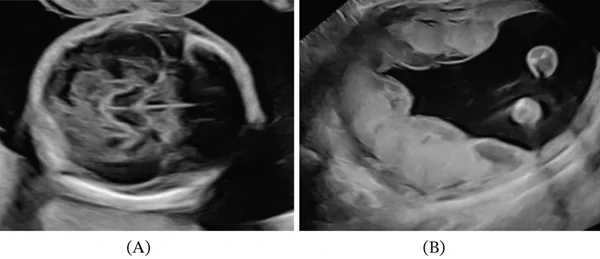
Anatomy ultrasound completed at 23 weeks of gestational age demonstrating normal cross section of (A) skull anatomy and (B) heterogeneous placenta.

### 2.1. Clinical Findings

The infant was born via a spontaneous vaginal delivery at 38 weeks, 5 days of gestation. 1‐ and 5‐min Apgar scores were 8 and 9, respectively, and birth weight was 3254 g. Initial exam was consistent with cephalohematoma overlying the right parietal bone, with a boggy sensation and “popping feeling” with palpation posterior to the right ear. Neurologic exam was normal with no deficits and normal spine. The timeline is illustrated in Figure 2.

**Figure 2:**
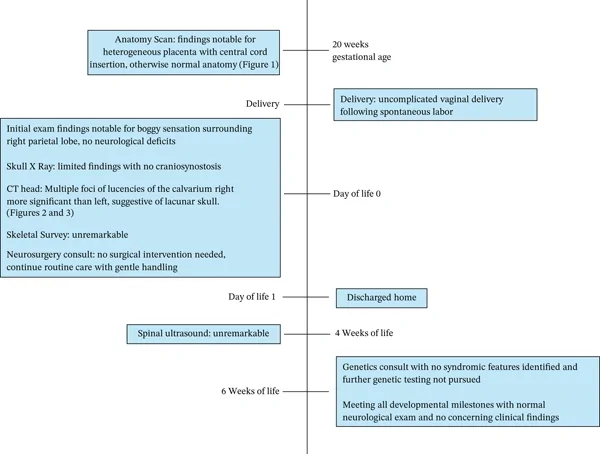
Timeline of fetal and neonatal assessments.

### 2.2. Diagnostic Assessment

On day of life 0, skull radiographic films were obtained although findings were limited and inconclusive given limited visualization of the calvarium. CT imaging with three‐dimensional reconstruction demonstrated multiple asymmetric lucent defects throughout the calvarium, more pronounced within the right parietal and occipital bones, consistent with lacunar skull (Figures 3 and 4) Brain parenchyma, cortical sulcation, ventricular size, and cranial sutures were normal. No evidence of hydrocephalus, Chiari malformation, craniosynostosis, intracranial hemorrhage, or other intracranial abnormalities was identified. The infant demonstrated a normal neurological exam.

**Figure 3:**
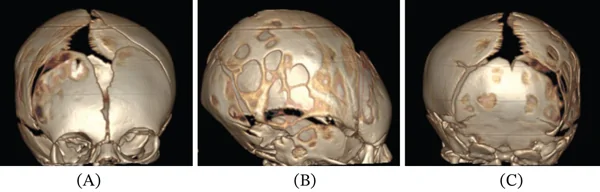
Three‐dimensional reconstruction of CT scan of the head of a 1‐day‐old female infant showing a (A) frontal view, (B) right side view, and (C) occipital view, demonstrating multiple lacunae diffusely along the right calvarium.

**Figure 4:**
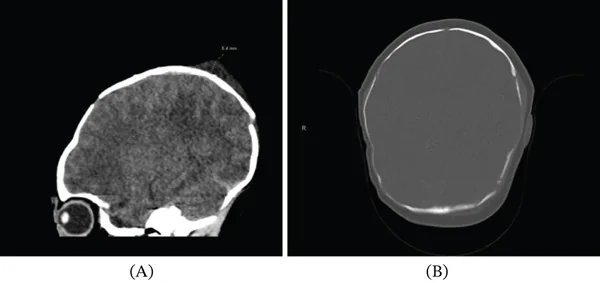
Noncontrast CT scan of the head of a 1‐day‐old female infant with (A) sagittal view and (B) axial view showing posterior parietal cephalohematoma with normal gyri and sulci.

Given the well‐recognized association between lacunar skull and other congenital disorders, additional evaluation was undertaken. Skeletal survey, spinal ultrasonography, and thyroid function studies were all within normal limits. The differential diagnosis included birth‐related skull fracture, skeletal dysplasia, osteogenesis imperfecta, and other congenital calvarial abnormalities. These diagnoses were considered unlikely based on the imaging findings, normal skeletal survey, absence of additional skeletal abnormalities, and otherwise normal physical examination.

### 2.3. Therapeutic Intervention

Given the absence of associated intracranial abnormalities or neurologic defects, conservative management with gentle handling and routine follow‐up was recommended by the neurosurgery team. The genetics team clinically evaluated the patient and did not identify any other findings suggestive of a syndromic disorder. Given the isolated nature of the lacunar skull and otherwise unremarkable diagnostic evaluation, genetic testing was unlikely to yield diagnostic results or change immediate clinical management. The family was counseled regarding the option for future genetic evaluation should any additional concerns arise.

### 2.4. Follow‐Up and Outcomes

The infant, now 6 weeks old, is doing well and meeting developmental milestones thus far.

## 3. Discussion

Lacunar skull is characterized by multiple oval or round translucent defects in the skull, resulting in a honey‐comb appearance on radiograph or CT scan [9]. Prior case studies have shown association of lacunar skull with craniosynostosis, neural tube defects and scoliosis [1–6]. Unlike the predominance of literature, our case is a presentation of lacunar skull without other identifiable pathology or comorbidities, adding to the limited existing data on this pathology. Previously published case reports evaluating isolated lacunar skull, two in 1981 [2, 10], and one from 2023 [5], are beneficial to the literature but do not offer clinical workup or postnatal findings of the neonate. There is therefore limited understanding of the natural history or long‐term clinical significance of isolated lacunar skull. The etiology of lacunar skull is still largely unknown. A potential explanation proposes that lacunar skull in cases of neural tube defects may result from an absence of cerebral ventricular distension secondary to decompression of an open neural tube [11]. This diminished intraventricular pressure is believed to impair the normal expansion of the fetal calvarium, thereby disrupting the typical development of membranous bone plates. As a result, abnormal collagen fiber accumulations may form, which produce the characteristic radiologic features observed in lacunar skull [11]. Despite this hypothesis, the etiology for isolated lacunar skull is still unexplained. While most lacunar skulls resolve through typical bone remodeling during childhood, some studies suggest ongoing or even worsening of lacunae throughout adolescence and into adulthood [6]. This persistence of lacunar skull has been associated with more frequent pathology such as scoliosis or Chiari malformation [6].

There is a paucity of literature on surgical management of lacunar skull in the absence of other surgically treated comorbidities like neural tube defects or craniosynostosis. Infants have the capacity to ossify large cranial defects due to signaling between underlying juvenile dura and cranial osteoblasts which persists until 2 years of age [12]. Thus, one could conclude that surgical management, for example with cranioplasty, of isolated lacunar skull in the perinatal period is not necessary. Further, the characteristic involvement of the inner table in lacunar skull would render autologous reconstruction with split calvarial bone grafting, the gold standard for cranioplasty in pediatric patients, difficult if not impossible [13]. Finally, the finding of persistent lacunar skull in patients up to 16 years of age without neurological manifestations suggests that surgical management may not be necessary. Further research is needed to determine best practice for radiologic monitoring and indications for surgical management as affected patients age.

Currently, most diagnoses of lacunar skull are made postnatally with CT with 3D reconstruction or x‐ray films [14]. In the current literature, no cases of intrauterine diagnosis are described, although suspicion is raised if radiologic findings of related comorbidities like neural tube defects are present, such as the classic “lemon sign” [15]. In prior case reports, the presence of lacunar skull during routine antenatal ultrasonography went undetected, as in our case presented [14]. However, fetal ultrasound continues to advance, with the addition of 3D rendering of the skeleton, and the integration of artificial intelligence for image optimization and diagnostics [16].

Further research is needed to identify potential ultrasonographic markers indicative of lacunar skull. Such findings would facilitate more comprehensive prenatal counseling and informed delivery planning by obstetric providers. Although a single published case report described fetal injury following operative vaginal delivery in an infant with lacunar skull [9], the available evidence is insufficient to determine whether suspected lacunar skull should influence delivery planning. Additional studies are needed before recommendations regarding operative vaginal delivery or mode of birth can be made Nonetheless, it is important for obstetricians to be aware that lacunar skull can also be associated with fetal neural tube defects, Chiari malformation, or neonatal scoliosis. Ongoing studies on the long‐term prognosis of isolated lacunar skull are essential to support informed counseling and optimal care strategies for affected individuals and their families.

This case represents a rare instance of isolated lacunar skull without associated anomalies, diagnosed postnatally despite normal prenatal imaging. It adds to the limited number of case reports of isolated lacunar skull, which we hope will be able to eventually build enough data for guiding diagnosis and care in pregnancy. It highlights the need for improved prenatal detection and further research to understand the condition′s cause, aiding better counseling and management.

## Funding

No funding was received for this manuscript.

## Disclosure

All authors have read and approved the final version of the manuscript. Dr. Mary Sterrett had full access to all of the data in this study and takes complete responsibility for the integrity of the data and the accuracy of the data analysis. Dr. Grace Noonan affirms that this manuscript is an honest, accurate, and transparent account of the study being reported; that no important aspects of the study have been omitted; and that any discrepancies from the study as planned have been explained.

## Consent

Informed consent was given by the patient for this study.

## Conflicts of Interest

The authors declare no conflicts of interest.

## Patient Perspective

From a patient perspective, the mother states she has barely noticed the lacunae by touch. Her daughter continues to appear healthy and is progressing as expected, and at no point has her child shown any discomfort when being held or touched.

## Supporting information


**Supporting Information** Additional supporting information can be found online in the Supporting Information section.

## Data Availability

Data sharing is not applicable to this article as no datasets were generated or analyzed during the current study.
