# Supplementary material for: Hidden Hollows of the Fetal Cranium—Lacunar Skull in Isolation
Source: Case Rep Obstet Gynecol. 2026 Sep 29;2026:8926011. doi: 10.1155/crog/8926011 (PMC13624703; doi:10.1155/crog/8926011)
Supplement: Supplementary file 1 [file CROG-2026-8926011-s001.pdf]

ELMER PRESS

Case Report Guidelines

(With reference to the CARE case report guidelines)

For fully describing case(s) and literature review, the manuscript word count should be more than 2500.

******Indicates required headings and subheadings*

***1 Title**

***2 Author(s)**

***3 Abstract (one paragraph, unstructured, up to 350 words)**

The patient's main concerns and important clinical findings;
The primary diagnoses, interventions, and outcomes;
What is unique about this case and what does it add to the scientific literature;
One or more learning points from this case report.

***4 Keywords (up to 10)**

***5 Introduction**

Briefly summarizes the background of this disease, and why this case is unique and may include medical literature references.

***6 Case Report**

This section **should** include **4** subheadings:

*1) Investigations (patient information and clinical findings)

De-identified patient specific information;
Primary concerns and symptoms of the patient;
Medical, family, and psychosocial history including relevant genetic information;
Relevant past interventions and their outcomes;
Significant physical examination (PE) and important clinical findings.
A Timeline is preferable: Historical and current information from this episode of care organized as a timeline (figure or table).

*2) Diagnosis

Diagnostic methods (PE, laboratory testing, imaging, surveys);
Diagnostic challenges;
Diagnosis (including other diagnoses considered);
Differential diagnosis if applicable;
Prognostic characteristics when applicable.

*3) Treatment

Types of therapeutic intervention (pharmacologic, surgical, preventive);
Administration of therapeutic intervention (dosage, strength, duration);
Changes in therapeutic interventions with explanations.

*4) Follow-up and outcomes

Clinician- and patient-assessed outcomes if available;
Important follow-up diagnostic and other test results;
Intervention adherence and tolerability, (How was this assessed?);
Adverse and unanticipated events.

*7 Discussion

Strengths and limitations in your approach to this case;
Discussion of the relevant medical literature;
The rationale for your conclusions.

*With a subheading of **Learning Points**, the primary "take-away" lessons (learning points) from this case report (without references) in one paragraph.

*8 Declarations

Acknowledgement
Financial Disclosure or Funding
Conflict of Interest
Informed Consent
Author Contributions
Data Availability

*9 References
